# Prevalence, Intensity, and Correlates of Soil-Transmitted Helminth Infections among School Children after a Decade of Preventive Chemotherapy in Western Rwanda

**DOI:** 10.3390/pathogens9121076

**Published:** 2020-12-21

**Authors:** Joseph Kabatende, Michael Mugisha, Lazare Ntirenganya, Abbie Barry, Eugene Ruberanziza, Jean Bosco Mbonigaba, Ulf Bergman, Emile Bienvenu, Eleni Aklillu

**Affiliations:** 1 Department of Laboratory Medicine, Division of Clinical Pharmacology, Karolinska Institutet, Karolinska University Hospital Huddinge,14186 Stockholm, Sweden; josephkabatende@gmail.com (J.K.); abbie.barry@ki.se (A.B.); ulf.bergman@ki.se (U.B.); 2 Rwanda Food and Drugs Authority, Nyarutarama Plaza, KG 9 Avenue Kigali, Rwanda; ntirenganyal1@gmail.com; 3 College of Medicine and Health Sciences, University of Rwanda, KK 737 Kigali, Rwanda; mmugisha@nursph.org (M.M.); ebienvenu3@gmail.com (E.B.); 4 Neglected Tropical Disease and Other Parasitic Disease Unit, Rwanda Biomedical Center, KG 17 Ave Kigali, Rwanda; ruberanzizaeugene@gmail.com (E.R.); jbosco.mbonigaba@rbc.gov.rw (J.B.M.)

**Keywords:** mass drug administration, preventive chemotherapy, neglected tropical diseases, intestinal soil-transmitted helminths, prevalence, school children, Rwanda

## Abstract

Preventive chemotherapy (PC) is a WHO-recommended core intervention measures to eliminate Soil-Transmitted Helminths (STH) as a public health problem by 2020, defined as a reduction in prevalence to <1% of moderate or high-intensity infection. We conducted a cross-sectional study to investigate the prevalence, intensity, and correlates of STH after a decade of PC in Rwanda. A total of 4998 school children (5–15 years old) from four districts along Lake Kivu in the western province were screened for STH using Kato-Katz. The overall prevalence of Soil-transmitted helminths among school children was 77.7% (range between districts = 54% to 92%). *Trichirus trichiura* was the most common STH (66.8%, range between districts = 23% to 88.2%), followed by *Ascaris lumbricoides* (49.9%, range between district = 28.5% to 63.3%) and hookworms (1.9%, range between districts = 0.6% to 2.9%). The prevalence of single, double and of triple parasite coinfection were 48.6%, 50.3%, and 1.1%, respectively. The overall prevalence of moderate or high-intensity infection for *Trichirus trichiura* and *Ascaris lumbricoides* was 7.1% and 13.9, respectively. Multivariate logistic regression model revealed that male sex, district, stunting, and schistosomiasis coinfection as significant predictors of STH infection. Despite a decade of PC implementation, STH remain a significant public health problem in Rwanda.

## 1. Introduction

Soil-transmitted helminth (STH) infections are the most common infections of neglected tropical diseases (NTDs) worldwide, primarily affecting the poorest and most deprived communities [1]. STHs are endemic in the tropical and sub-tropical regions, with the highest burdens occurring in sub-Saharan Africa, the Americas, China, and East Asia [2]. More than 1.5 billion or 24% of the world’s population suffer from one or more STH infections [1]. The most common species of STH that infect people are the roundworm (*Ascaris lumbricoides*), the whipworm (*Trichirus trichiura*), and various species of hookworms (*Necator americanus Ancylostoma duodenale* and *Ancylostoma ceylanicum*) [1]. *Ascaris* and *Trichirus* infections are transmitted through contaminated food and water, whereas hookworms are transmitted by skin penetration [3]. Children are by far the most disproportionately affected by STH, and infected children are usually malnourished and anemic because of the resultant nutritional deficiency. Over 568 million school-age children live in areas where these parasites are intensively transmitted [1]. Numerous studies have indicated that poor water, sanitation, and hygiene (WASH) put children at higher risk of STH infections [3,4]. STH infection contributes to delayed intellectual development, diminished physical fitness, growth retardation, and cognition [5].

To reduce the morbidity of schistosomiasis and STH, in May 2001, the World Health Assembly endorsed a resolution for regular treatment of high-risk groups, particularly school-age children, through mass drug administration (MDA) of anthelmintics [6]. Large-scale preventive chemotherapy (PC) (also called deworming) through periodic mass administration of single-dose albendazole 400 mg or mebendazole 500 mg to at-risk population is WHO’s core intervention strategy to control morbidity [7]. The WHO intervention program targets to eliminate STH as a public health problem (defined as a reduction in prevalence to <1% of moderate or high-intensity infection) by 2020 [8]. Although the WHO intervention strategy recommends PC to all at-risk groups, NTD programs in many endemic countries, including Rwanda, provides MDA to pre-school children (Pre-SAC) and school-aged children (SAC), while adults-at-risk are not considered and may serve as a source of infection to treated children [9].

Following the WHO recommendations, school-based MDA has been initiated in many endemic countries, including Rwanda. The national NTD control program in Rwanda was established in 2007. The first MDA was delivered in 2008 after the initial disease mapping pre-intervention survey that revealed that >65% of children had intestinal worms with high levels of multiple parasite coinfection [10]. Although Rwanda achieved about 100% coverage of albendazole and praziquantel MDA, mostly targeting school-aged children in 2008–2010, the transmission of *Schistosoma mansoni (S. mansoni*) and STHs continued as reported by a follow-up survey [9]. The mapping report of 2014 revealed that the average STHs prevalence in all districts was 45.2% compared to 65.8% that was reported by the pre-intervention mapping in 2008; this shows a decrease in the prevalence of 20.6% countrywide [11]. However, no assessment was done on the effectiveness of long-term interventions in controlling morbidity. Continued surveillance after a decade of program implementation is important to assess the STH control program’s impact and determine the next steps [12]. As the milestone of WHO target to eliminate STH as a public health problem in 2020 is due, surveillance data assessing the impact of long-term PC in reducing the disease burden is critical for evidence-based decision making. The aim of the study was to investigate the prevalence, infection intensities, and factors associated with STH among school children in four rural districts in the western province of Rwanda.

## 2. Results

### 2.1. Prevalence and Intensities of STH Infections

Out of the 4998 screened children, 3885 were infected with one or more STH parasites giving an overall prevalence of 77.7% (95% CI = 76.6 to 78.9%) STH infection. There was a significant difference in prevalence of STH infection between the 4 study districts, being highest in Rubavu (92%) and Ruszi districts (89%) compared to Nyamasheke (60%) and Rutsiro (54%). *T. trichiura* was the most prevalent STH infection, followed by *A. lumbricoides*. The overall prevalence of *T. trichiura* infections was 66.8% (3338/4998; 95% CI = 65.5 to 68.1%), and that of *A. lumbricoides* infections was 49.9% (2495/4998; 95% CI = 48.5 to 51.3%). The overall prevalence of hookworms was 1.9% (93/4998; 95% CI = 1.5 to 2.3%). The overall prevalence and infection intensity is presented in Table 1. The intensity of infection was defined for each parasite as ‘‘light’’, ‘‘moderate’’ or ‘‘heavy’’ based on fecal egg counts per gram of stool (epg) using the cut-off threshold set by WHO [13] as follows:

○ *T. trichiura*: Light (1–999 epg), Moderate (1000–9999 epg), Heavy (≥10,000 epg).
○ *A. lumbricoides*: Light (1–4999 epg), Moderate (5000–49,999 epg), Heavy (≥50,000 epg).
○ Hookworm: Light (1–1999 epg), Moderate (2000–3999 epg), Heavy (≥4000 epg).

### 2.2. Prevalence of Single and Multiple STH Parasite Coinfections

**Figure 1:**
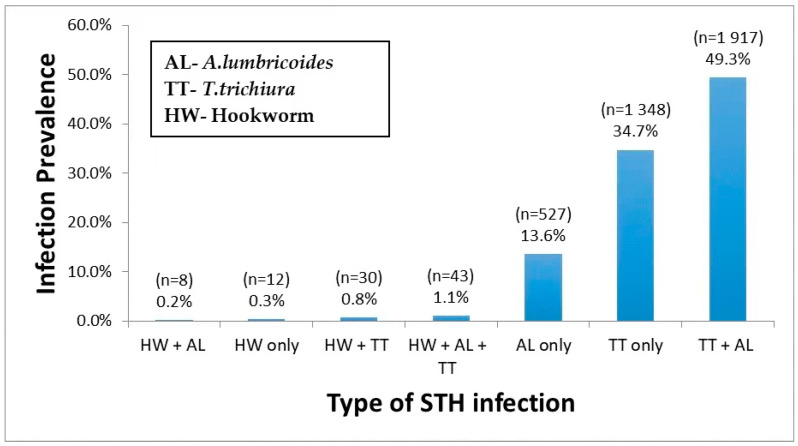
Prevalence of single, dual, and triple STH parasites coinfections.

Out of 3885 STH infected children, 48.6% (1887/3885) had a single helminth infection, either with *T. trichiura* (34.7%) or *A. lumbricoides* (13.6%) or hookworm (0.3%) only. The prevalence of multiple parasite coinfection was 50.3%, the most common dual infection by *T. trichiura* and *A. lumbricoides* (49.3%). Triple infections prevalence *T. trichiura, A. lumbricoides*, and hookworm was 1.1%. Among STH infected children, 4.7% were coinfected with schistosomiasis. The prevalence and type of single, double, and triple infections stratified by the type of STH species involved are presented in Figure 1.

### 2.3. Change in Prevalence of STH Infection Overtime among Study Districts

**Figure 2:**
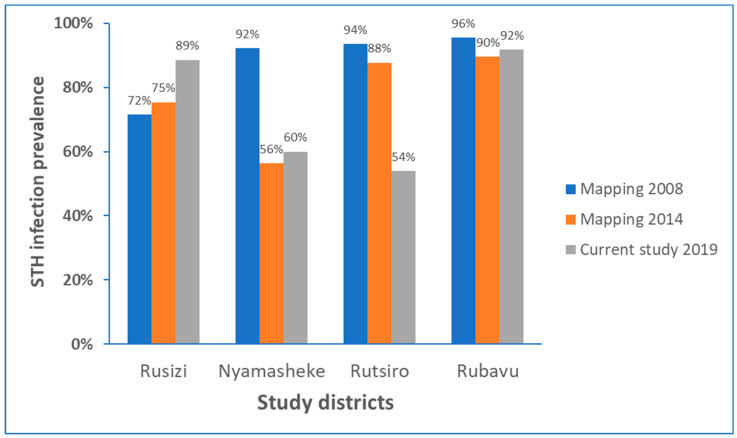
STH infection prevalence before starting preventive chemotherapy in 2008, and follow-up in 2014, and the current study in 2019 in each study district.

Comparison of prevalence of STH infection identified the current study (conducted in 2019), with respective STH mapping data from 2008 conducted before starting MDA intervention (baseline) STH [10,12], and the follow-up STH mapping conducted in 2014 [11] for each study districts is presented in Figure 2. There was no significant impact of MDA implanted during the past ten years in reducing the burden of STH, particularly in Rubavu (92%) and Rusizi (89%), the most highly STH affected districts in this study. In the Rutsiro district, the STH prevalence has reduced by 35% from the baseline data, whereas in Nyamasheke, the reduction in STH burden observed in 2014 is leveled off in 2019.

**Figure 3:**
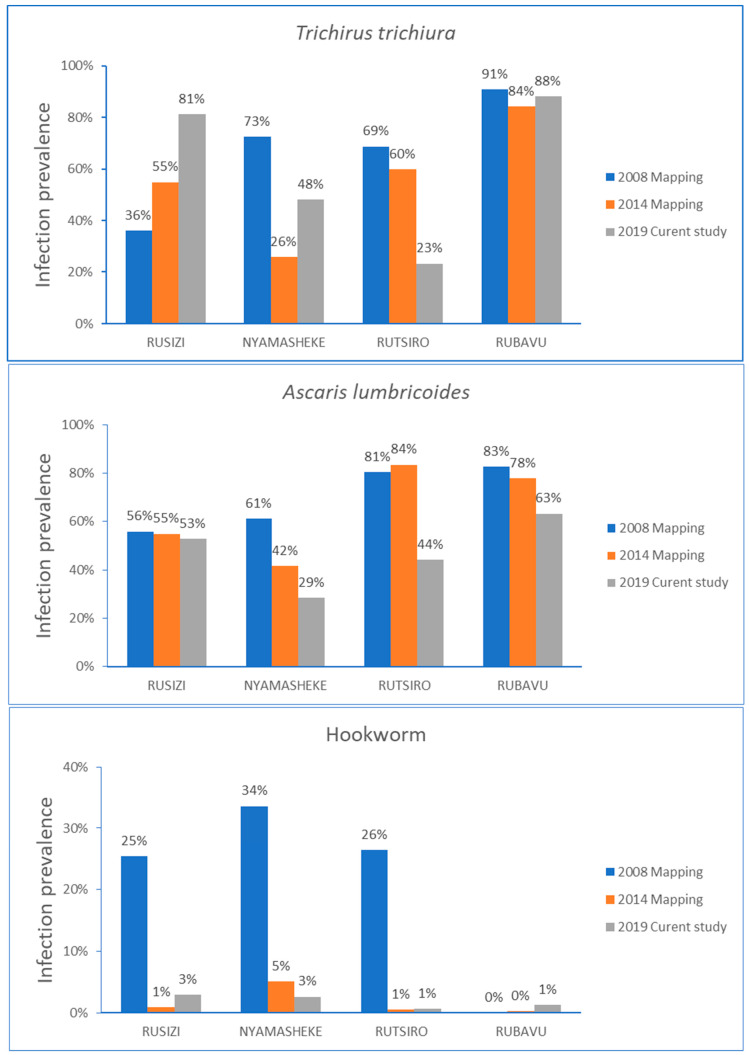
Comparison of STH infection prevalence before starting MDA in 2008, follow-up mapping in 2014, and the current study in 2019 stratified by type of parasite for each study district.

The comparison of STH infection prevalence over time stratified by type of parasite infection for each study district is presented in Figure 3. In the Rusizi district, the STH infection prevalence steadily increased from the baseline data collected in 2008, mainly for *T. trichiura* infection. Infection with A. *lumbricoides* gradually decreased in all districts, except in Rusizi, where no change in the infection prevalence over time was observed. The hookworm infection prevalence rate reduced significantly from the baseline data for Rusizi, Nyamashele and Rutsiro, and the prevalence of hookworm remained low in the Rubavu district.

### 2.4. Correlates of STH Infections

Any correlations of sociodemographic characteristics and nutritional status determined using the WHO anthropometric measurement standards with STH infection were analyzed (Table 2). Anthropometric measurements were converted to height for age Z score (HAZ) and body mass index (BMI) for age Z score (BAZ) using the WHO Anthro-Plus software version 1.0.4 [14]. Children with HAZ and BAZ scores less than two standard deviations were classified as stunted and wasted/thin, respectively. STH infection was significantly correlated with sex, age group, living district, school, stunting, and wasting. Prevalence of STHs infection were significantly higher in males than females for all STH species except hookworms. The prevalence of STHs infection were also significantly different between districts and schools. Rubavu and Ruszi districts were the most affected by STH infection, where 91.8% and 88.6% of the children were infected by at least one or more STH parasite species. Rambo and Rubona schools (both located in Rubavu districts) were the most affected, where 93% and 92% of the children were infected by at least one or more STH species. Stunting was significantly associated with at least one or more STH infections.

### 2.5. Risk Factors Associated with Infection Intensity

A negative binomial regression model was used to identify factors associated with infection intensity (eggs count/gram of stool) among the study population (Table 3). Using negative binomial regression, living in Nyamasheke and Rusizi districts were the only significant risk factors for having higher hookworm infection intensity. Stunting and districts were significant risk factors for higher infection intensity for *A. lumbricoides*. Similarly, age (*p* = 0.001), district (*p* < 0.001), and stunting (*p* = 0.006) were significantly associated with higher *T. trichiura* infection intensity. Sex differences and wasting status were not significant risk factors for high infection intensity. Likewise, no significant effect of age with infection intensity was observed, except for *T. trichiura*, where the higher age group (10–15 years old) had increased risk for high infection-intensity for *T. trichiura* compared to the low age group (5–9 years old).

### 2.6. Risk Factors Associated with Any STH Infections

A univariate and multivariate logistic regression analysis of factors associated with STH infections is presented in Table 4. On the univariate logistic regression model, being male was significantly associated with any STH infection (OR: 1.27, 95% CI: 1.11–1.46). Living in the district of Rubavu, Nyamasheke and Rusizi were significantly associated with STH infection in reference to Rutsiro district, with the lowest STH infection prevalence (OR: 9.5, 95% CI: 7.69–11.84), (OR: 1.27, 95% CI: 1.07–1.53), and (OR: 6.6, 95% CI: 5.32–8.25]) respectively. Studying at the following schools were significantly associated with STH infection: Rambo (OR: 11.5, 95% CI: 8.29–16.1), Rubona (OR: 8.5, 95% CI [6.37–11.42], Buhokoro (OR; 1.37, 95% CI [1.05–1.77], Bugumira (OR: 5.89, 95% CI [4.24–8.20], and Nkombo (OR:7.37, 95% CI: [5.46–9.96] compared to studying at Sure primary school. Analysis with multivariate logistic regression model revealed that sex, district, stunting, and schistosomiasis coinfection remained predictors of STH infection.

### 2.7. Factors Associated with Hookworms Infection

Univariate and multivariate logistic regression for factors associated with hookworms, *A. lumbricoides* and *T. trichiura* is presented in Table 5. On univariate analysis, schistosomiasis coinfection and living in Rubavu, Nyamasheke and Rusizi districts were significantly associated with hookworm infection in reference to Rutsiro district with (OR: 2.17, 95% CI: 0.82–5.73), (OR: 4.47, 95% CI: 1.71–11.64), and (OR: 5.07, 95% CI: 1.98–12.96) respectively. Schooling at Buhokoro and Nkombo Primary school were significantly associated with hookworm infection in reference to schooling at Sure Primary school with (OR: 6.36, 95% CI: 1.47–27.58) and (OR: 7.22, 95% CI: 1.7–30.55) respectively. Being Stunted was significantly associated with hookworm infection (OR: 1.55, 95% CI: 1.02–2.34). Analysis with multivariate logistic regression model showed that living in Nyamasheke and Rusizi district, and schistosomiasis coinfection remained significant predictors of hookworm infection.

### 2.8. Factors Associated with Ascaris Lumbricoides Infection

The univariate logistic regression model indicated that being male children were significantly affected with *A. lumbricoides* infection (OR 1.15, 95% CI: 1.04–1.29) compared to female children (Table 5). Living in Rubavu, Nyamasheke and Rusizi districts were significantly associated with *A. lumbricoides* infection in reference to Rutsiro district with (OR: 2.18, 95% CI: 1.84–2.57), (OR: 0.5, 95% CI: 0.41–0.61) and (OR: 1.42, 95% CI: 1.19–1.69) respectively. Studying at Rambo, Rubona, Buhokoro, Mukoma, Bugumira and Nkombo schools were significantly associated with *A. lumbricoides* infection compared to studying at Sure primary school with (OR: 2.84, 95% [2.22–3.62]), (OR: 1.92, 95% [1.52–2.44]), (OR: 1.1, 95% CI [0.84–1.45]), (OR: 1.41, 95% CI [1.1–1.84] and (OR: 1.54, 95% CI [1.21–1.96] respectively. Being stunted was significantly associated with *A. lumbricoides* infection. Analysis with multivariate logistic regression model showed that being male child, living in Rubavu, Nyamasheke, Rusizi district and being stunted remained significant predictors of *A. lumbricoides* infection.

### 2.9. Factors Associated with Trichuris Trichiura Infections

On univariate logistic regression model, being male child were significantly associated with *T. trichiura* infection (OR: 1.16, 95% CI: 1.04–1.31) (Table 5). Living in Rubavu, Nyamasheke and Rusizi district were significantly associated with *T. trichiura* infection in reference to Rutsiro district with (OR: 24.65, 95% CI: 19.89–30.54), (OR: 3–06, 95% CI: 2.51–3.74), and (OR: 14.2, 95% CI: 11.48–17.57) respectively. Studying at Rambo, Rubona, Buhokoro, Mukoma, Bugumira and Nkombo were significantly associated with *T. trichiura* infection compared to Sure Primary school with (OR: 25.1, 95% CI: 18.3–34.3), (OR: 20.2, 95% CI: 15.1–27.1), (OR: 2.9, 95% CI: 2.17–3.78), (OR: 2.6, 95% CI: 1.98–3.51), (OR: 12, 95% CI: 8.74–16.48) and (OR: 13.3, 95% CI: 9.99–17.82). Being stunted was significantly associated with *T. trichiura* infection (OR: 1.32, 95% CI: 1.17–1.51 and wasting was significantly protecting factor of *T. trichiura* infection (OR: 0.56, 95% CI: 0.44–0.71). Analysis with multivariate logistic regression model, being aged from 10–15 years old, schistosomiasis coinfection, living in Rubavu, Nyamasheke, Rusizi district and being stunted remained significant predictors of *T. trichiura* infection.

## 3. Discussion

**Figure 4:**
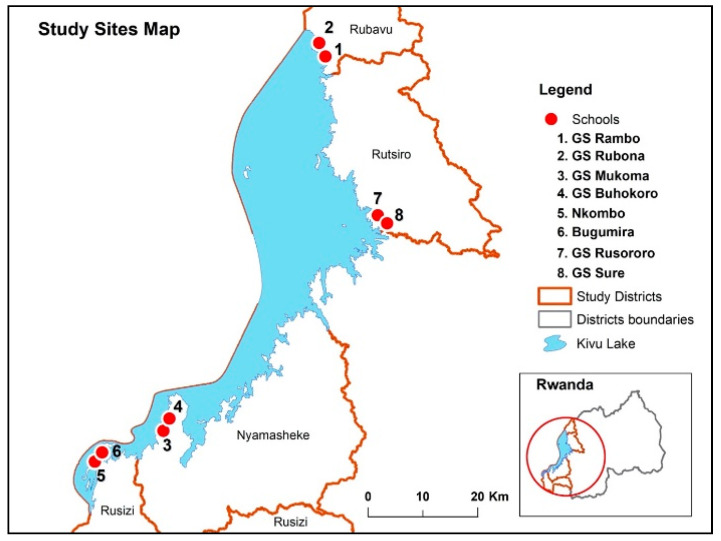
Map of Rwanda showing the study districts and selected schools.

This cross-sectional study investigated the prevalence, intensity, and associated risk factors for STH infections (ascariasis, trichuriasis and hookworms) among school children 5–15 years old attending eight primary schools in four rural districts in the Western province of Rwanda. The study districts (Rusizi, Nyamasheke, Rubavu, and Rutsiro) lay on the belt of lake Kivu (Figure 4), which is among the most STH endemic region in Rwanda. The main findings of the study include; (i) a high overall prevalence (77.7%) of STH infection, though prevalence significantly varies between study districts (ranging from 54% to 92%), and between study schools (ranging from 54% to 93%), (ii) *T. trichiura* (66.8%) was the most prevalent STH parasite species followed by *A. lumbricoides* (49.9%), and hookworms (1.9%), (iii) a high prevalence of multiple parasite coinfection (>50%) mostly with *T.trichiura* and *A. lumbricoides*, (iv) stunting, male sex, living district, school, and schistosomiasis coinfection were significant predictors of STH infection, (v) stunting and living district were significant predictors of high infection intensity, particularly for *T. trichiura* and *A. lumbricoides* infections. To our knowledge, this is the most extensive study to evaluate the status and predictors of STH infections after long-term implementation of multiple rounds of MDA in Rwanda and sub-Saharan Africa at large.

The study findings revealed that 32% of the children were stunted, which is in line with Rwanda Demographic and Health Survey’s national data, reporting 38% of children were stunted in 2015 [15]. A recent study from Tanzania [16] reported that 29% of schoolchildren were stunted. Our result indicates a significant correlation between stunting with having STH infection, regardless of parasite species involved (Table 2). Stunting was also a predictor of high infection intensity (high number of eggs counts/gram of stool), particularly for infection by *A. lumbricoides* and *T. trichiura*. Similar findings were reported from Uganda and Kenya, which revealed that children affected by STH are usually malnourished and anemic because of nutritional deficiency [17,18]. Stunting due to STH infection may contribute to delayed intellectual development, diminished physical fitness, growth retardation, and cognition [5,19]. The high prevalence of coinfection between *T. trichiura* and *A. lumbricoides* (49.3%) found in this study is consistent with a recent report that most of the dual STH infections observed in Ethiopia involve *A. lumbricoides* and *T. trichiura* [20]. In line with the report from Ethiopia [20], we also found a significant correlation between schistosomiasis and STH coinfection (Table 4). Our study revealed that sex, district, school, stunting, and schistosomiasis coinfection as significant predictors of STH infection.

Large-scale targeted preventive chemotherapy or deworming to at all risk-population is WHO’s core intervention strategy to control morbidity and eliminate STHs as a public health problem [8]. In 2008, the Rwandan national NTD program conducted pre-intervention disease mapping. The baseline survey reported that STH and schistosomiasis infection was a significant public health problem in Rwanda, where more than 65% of children had intestinal worms with high levels of multiple parasite coinfection prevalence [10]. Since 2008, Rwanda’s national NTD program has been implementing a targeted school-based biannual deworming program with albendazole and an annual deworming for praziquantel in all areas with a moderate-to-high prevalence rate of STH and schistosomiasis infections. Early follow-up surveys indicated that despite Rwanda achieved about 100% coverage of MDA during 2008–2010, the transmission of STHs continued [9]. The mapping report of 2014 revealed STH prevalence of 45.2% STH infection countrywide, which is a decrease by 20.6% from the 65.8% pre-intervention mapping report in 2008 [11].

The current study was conducted after ten years (in 2019) nationwide implementation of MDA in Rwanda, with >68.8 million and >3.2 million treatments delivered to children against STH and schistosomiasis, respectively [21]. The overall STH infection prevalence (77.7%) and the high multiple parasite infection prevalence (>50%) observed in this study indicate that STH infection remains a significant public health problem in Rwanda, despite a decade of preventive chemotherapy with nearly 100% coverage. This study can serve as a proxy to assess the impact of a 10-year preventive chemotherapy implementation to control and eliminate STH infection in the 4 study districts. Apparently, despite biannual MDA for years, the burden of STH infection, in particular *T. trichiura* infection, remain the same in Rubavu and paradoxically increasing in Ruszi district (Figure 2 and Figure 3). Nevertheless, the applied standard single-dose albendazole 400 mg preventative chemotherapy has successfully lowered the burden of hookworm infection and slightly reduced *A. lumbricoides* infection, but with no significant impact on *T. trichiura* infection. The significant decrease in hookworm infection prevalence rate over time compared to *A. lumbricoides* and *T. trichiura* could be due to variations in albendazole efficacy against the three parasite species. A systematic review and meta-analysis study reported the highest efficacy of albendazole against hookworm infection and a significant reduction in the efficacy of albendazole against *T. trichiura* over the past two decades [22]. Lack of efficacy of albendazole or mebendazole against *T. trichiura* is also reported previously [23,24]. A randomized clinical trial that investigated the efficacy of 200 mg, 400 mg, 600 mg of albendazole or placebo in pre-school children, and 400 mg, 600 mg, 800 mg of albendazole or placebo in school children reported that low efficacy against *T. trichiura* in both study populations and by all studied doses [25]. Search for other alternative treatment against *T. trichiura* infection is ongoing, including albendazole combination therapy with oxantel pamoate [26] or with moxidectin [27] but so far with limited success. On the other hand, reduced efficacy of albendazole against *A. lumbricoides* in Rwandan school children is reported previously [28]. Available reports, including ours, highlight that to eliminate STH as a public health problem in endemic regions, new drugs or alternative treatment regimens, particularly for *A. lumbricoides* and *T. trichiura is* urgently needed.

The study findings indicate that residence districts and schools are significant predictors of STH infection. The most affected districts were the Rubavu and Ruszi districts, where 91.8% and 88.6% of the children were infected by one or more STH species. Rambo and Rubona schools were the most affected, where 93% and 91% of the children were infected by at least one or more STH species (Table 2). A recent study conducted in Nkombo Island in the western province of Rwanda revealed a higher overall prevalence of STH (95.2%) infection, and the prevalence for *T. trichiura, A. lumbricoides*, and hookworm being 92.9%, 35.7%, and 11.6% for respectively [29] This further highlights a similar trend in STH species with the current study finding (prevalence of 67% for *T. trichiura*, 50% for *A. lumbricoides*, and 1.9% for hookworm). Available reports, including ours, indicate a considerable burden of STH in the western province of Rwanda.

The WHO intervention program set a goal to eliminate STH as a public health problem by 2020. For operation purposes, WHO defines STH infection as a public health problem when the prevalence of moderate or high-intensity in at-risk populations is >1% [8]. Although most children had light intensity infection, the overall prevalence of moderate or high-intensity infection for *T. trichiura* and *Ascaris lumbricoides* in our study was 7.1% and 13.9%, respectively (Table 1). This indicates that Rwanda is far from achieving the intended WHO target to eliminate STH as a public health problem by 2020. It is well recognized that the WHO intervention strategy and implementation of the global NTD program have contributed to reducing the disease burden. However, the long-term intervention measures taken so far by many endemic countries in sub-Saharan Africa, including Rwanda, have not managed to control and eliminate STH and other NTDs as a public health problem by 2020 [16,20,30,31]. Several factors may have contributed to this shortcoming [32]. With respect to STH infections, apart from increasing MDA coverage, adequate access to clean water, sanitation, and hygiene are vital to reduce the risk of parasite exposure and halt transmission [33,34]. In 2015, WHO recommended a global plan to better integrate water, sanitation, and hygiene (WASH) services with four other public health interventions to accelerate progress in eliminating and eradicating neglected tropical diseases (NTDs) by 2020 [35]. Although many endemic countries managed to achieve high MDA coverage, limited coordination with the water, sanitation, and hygiene (WASH) sector in many endemic countries remains challenging [32]. Recognizing this challenge, Rwanda revised its Neglected Tropical Diseases Strategic Plan 2019–2024 and set a goal to make Rwanda free from NTDs as a public health problem by 2024 through the implementation of WHO-recommended public health strategies for prevention and control of NTDs [21]. Recent expert review projects that with sustained program implementation, the global elimination of STH infections as a public health problem might be achieved by 2030 [36].

The other contributing factor for the high prevalence of STH in our study could be the reduced effectiveness of albendazole in killing STHs. The large-scale repeated use of anthelminthic drugs and repeated drug exposure may increase the likelihood of the targeted parasite developing resistance. Lack of impact of repeated mass albendazole chemotherapy in preventing *T. trichiura* transmission observed in this study, and the reduced efficacy of albendazole against *A. lumbricoides* in Rwandan school children reported previously [28] is a concern. Drug resistance should be suspected if high-frequency anthelminthic treatment with high-coverage is found to have less than the expected effect on the target parasite [37]. After four years of implementing PC programs for schistosomiasis and soil-transmitted helminthiases, anthelminthic drug efficacy assessment is recommended [37]. As the current mass STH control programs, rely almost exclusively on few available benzimidazole anthelmintics, regular surveillance and monitoring of drug efficacy are essential for early detection of parasite resistance so that mitigation strategies such as combination therapy to prolong the effectiveness of the existing anthelmintic drugs, and intensify the search for new anthelmintic drugs or intervention measures [38].

This study has some limitation. Although we used Kato-Katz, the most widely used diagnostic method recommended by WHO for epidemiologic surveys and anthelminthic drug efficacy studies against schistosomiasis and STH [37,39], its sensitivity decreases in low prevalence and low-intensity settings [40]. Low sensitivity of Kato-Katz for detection of hookworm infection may be related to rapid degeneration of delicate hookworm eggs with time [41]. To overcome this limitation, fresh fecal samples were processed and read onsite at each study school within one hour of sample collection. In addition, the current study was done in a similar set-up using the same diagnostic method (Kato-Katz) used previously by the national NTD program during the 2008 and 2014 STH mapping. Therefore the significant decline in hookworm prevalence in this study compared to the mapping data in 2008 and 2014 (Figure 3) could possibly be due to repeated preventive chemotherapy, since albendazole displays highest efficacy rate against hookworm than *A. lumbricoides* and *T. trichiura* [22]. Use of Kato-Katz method for STH screening is useful to define infection intensity according to the WHO threshold [13]. The prevalence of moderate or heavy infection intensities in at-risk population can serve as a surrogate marker to assess the impact of preventive chemotherapy in eliminating STH as a public health problem [8]. As our study was done in a resource-limited setting with relatively higher infection prevalence and intensity, we consider that our finding is valid despite using the less sensitive Kato-Katz technique. Nevertheless, the observed light intensity-infection prevalence in this study could possibly be increased by using a more sensitive molecular diagnostics methods such as quantitative polymerase chain reaction (qPCR).

## 4. Materials and Methods

### 4.1. Study Area, Population, and Design

The descriptive cross-sectional study was conducted in April 2019 among school children attending eight selected schools in the four selected districts in the western province of Rwanda. The western province has an area of 4724.8 km^2^ covered by land, forests, and large water bodies, especially Lake Kivu. The province has seven administrative districts and ninety-six sectors and is inhabited by 2,471,239 inhabitants. Four districts around the belt of lake Kivu namely, Rubavu, Rutsiro, Nyamasheke, and Rusizi, were selected for this study based on epidemiological data related to STH. Most inhabitants in the selected districts carry out their daily activities in close contact with water bodies such as fishing, farming, bathing, washing, and swimming in Lake Kivu. The proximity of selected schools was located about five kilometers from the Lake. The map below indicates the study site and proximity to the water body.

The four districts in the western province were selected using a purposive sampling method based on STH prevalence data from previous studies [9,11]. Within each district, two schools were selected based on three criteria: (1) proximity to the lakes, (2) the number of school children attending, and (3) previous STH prevalence data. A sample proportion of each school to contribute to the whole study sample was based on each student population size. This was distributed to classes, and schoolchildren were systematically sampled in each class using class lists. A total of 5000 children aged 5–15 years from eight selected schools were pre-screened to determine their STH status.

This cross-sectional study enrolled a total of 4998 school children (5–15 years old) attending eight primary schools in four rural districts of Rwanda’s western province. Sociodemographic characteristics of study participants are presented in Table 6. The nutritional status of screened children indicated that 1599 (32%) were stunted and 302 (6%) were wasted.

### 4.2. Inclusion and Exclusion Criteria

Schoolchildren aged between 5–15 years attending the selected schools whose parents or guardians gave written informed consent for their participation and provided ascent to participate were included. Schoolchildren whose parents or guardians were not willing to provide informed consent and or not assent were not included in the study.

### 4.3. Data Collection

School children and their parents/guardians were first informed on the purpose of the study and the process of sample collection. Sociodemographic and anthropometric data were collected using a case record format prior to stool sample collection. Trained data collectors collected demographic information and anthropometric measures supported by trained schoolteachers. A complete participant form with a unique identification number, and stool sample was handed to the laboratory technician to screen and complete laboratory exam results. The data collector immediately entered a completed questionnaire in the electronic database-using tablets. A data manager reviewed submitted data daily to cross-check and rectify for errors, incomplete and/or missing data. The study coordinator assigned for each school supervised the study enrolment, data collection, and entry to the database, and laboratory analysis process. The bodyweight of children was measured in kilogram (Kg) and height in centimeter (cm). For measurement accuracy, the weighing scale was calibrated daily.

### 4.4. Screening for STH Parasite Species

Stool samples were collected from all study participants, and two Kato Katz smears were prepared from the collected stool sample using a template of 41.7 mg and processed as described previously [13,42]. Duplicate slides were prepared from each stool sample and read independently by the two laboratory technicians. Lab technicians from the National Reference Laboratory, Hospitals and Health Centers analyzed samples, and senior lab technicians were designated to conduct quality control and analyzed up to 10% of all stool samples examined per day. Before data collection, all lab technicians were trained and supervised by research coordinators.

### 4.5. Data Processing and Statistical Analysis

All data collected in the electronic database were imported in STATA 13 for cleaning and analysis. Outcome variables were categorized as positive and negative for any STH parasite infection as well as stratified by individual species (hookworm, *A. lumbricoides*, and *T. trichiura*). A participant was positive with any species when he or she had at least one egg count of that species on one of two slides tested. A participant was considered negative when he or she had 0 egg count of that species on both slides tested. A participant was considered STH positive when he/she was positive for one of the species investigated (hookworms, *A. lumbricoides*, and *T. trichiura*).

Associations between a categorical dependent variable and independent categorical variables were analyzed using the Chi-square test. Factors associated with egg count/gram of stool were analyzed using a negative binomial regression model. We used log transformation to change negative binomial regression coefficients into Incidence Risk Ratios (IRR) for interpretation without changing their estimations. Predictors of any STH infection and infection by specific species (Hookworms, *A. lumbricoides* and *T. trichiura*) were first analyzed using univariate followed by multivariate binary logistic regression models. Predictors with *p* ≤ 0.1 in the univariate analyses were entered in multivariate analysis using a forward and backward stepwise regression model. A *p*-value of less than 0.05 was considered statistically significant.

### 4.6. Ethical Consideration

This study was approved by the Rwandan National Ethics committee (Review Approval Notice: No 0064/RNEC/2019) and National Health Research Committee of the Ministry of Health, Rwanda (NHRC/2018/PROT/042. Before initiating the study, an awareness creation and sensitization meeting was held with education offices at the district and head of district hospitals, health centers, schoolteachers, school administrators and parents/guardians. Prior to enrolment, participants were explained the purpose and the conduct of the study, and a written informed consent from parents/guardians as well as assent from participating school children were obtained.

## 5. Conclusions

We report a high overall prevalence of STH infection, and multiple STH parasite coinfection among school children living the study area. Infection by *T. trichiura* is the most prevalent, followed by *A. lumbricoides*. Although the prevalence and infection-intensity vary between study districts and schools, the finding of >90% of children infected in the most affected districts and schools is worrisome. After a decade of program implementation with high MDA coverage, the observed 20% total prevalence of moderate or high-intensity infection indicates that Rwanda is far from achieving the intended WHO target of <1% prevalence to eliminate STH as a public health problem by 2020. Intensified control measures, including improved access to clean water, hygiene, and sanitation, need to integrate along with regular MDA to achieve the revised Rwanda NTD strategic plan goals-to make Rwanda free from NTDs as a public health problem by 2024. A multisectoral approach that brings together policymakers, researchers, regulators, the national NTD program, and the community is vital to control morbidity and elimination of STH in Rwanda.

## Figures and Tables

**Table 1:** Prevalence of STHs infections stratified by infection intensities for each STH parasite.

Parasite	Infection Prevalence	Intensity of Infection			
		No Infection	Light	Moderate	Heavy
***Any STH parasit***	77.7%				
***T. trichuria***	66.8%	33.2%	59.7%	6.9%	0.2%
***A. lumbricoides***	49.9%	50.1%	36.1%	12.8%	1.1%
**Hookworms**	1.9%	98.1%	1.8%	0	0

**Table 2:** Associations between demographic characteristics and STH among school children (*n* = 4998).

Variable		STH Positive (*n* = 3885)		*T. trichiura* (*n* = 3338)		*A. lumbricoides*. (*n* = 2495)		Hook Worms (*n* = 93)	
		*n* (%)	*p*-Value	*n* (%)	*p*-Value	*n* (%)	*p*-Value	*n* (%)	*p*-Value
Sex			<0.001		0.01		0.009		0.74
	Male	1874 (80.0)		1608 (68.6)		1216 (51.9)		42 (1.8)	
	Female	2011 (75.8)		1730 (65.2)		1279 (48.2)		51 (1.9)	
Age categories			0.53		0.18		0.66		0.05
	5–9 years	1183 (77.2)		1003 (65.4)		758 (49.5)		20 (1.3)	
	10–15 years	2702 (78.0)		2335 (67.4)		1737 (50.1)		73 (2.1)	
District			<0.001		<0.001		<0.001		<0.001
	Rubavu	1659 (91.8)		1594 (88.2)		1144 (63.3)		23 (1.3)	
	Rutsiro	457 (54.0)		197 (23.3)		374 (44.2)		5 (0.6)	
	Nyamasheke	649 (60.0)		521 (48.2)		308 (28.5)		28 (2.6)	
	Rusizi	1120 (88.6)		1026 (81.2)		669 (52.9)		37 (2.9)	
School			<0.001		<0.001		<0.001		<0.001
	Rambo	773 (93.0)		743 (89.4)		566 (68.1)		12 (1.4)	
	Rubona	886 (90.8)		851 (87.2)		578 (59.2)		11 (1.1)	
	Rusororo	240 (54.4)		95 (21.5)		200 (45.4)		3 (0.7)	
	Sure	217 (53.6)		102 (25.2)		174 (43.0)		2 (0.5)	
	Buhokoro	360 (61.2)		289 (49.2)		169 (28.7)		18 (3.1)	
	Mukoma	289 (58.6)		232 (47.1)		139 (28.2)		10 (2.0)	
	Bugumira	421 (87.2)		387 (80.2)		249 (51.7)		10 (2.1)	
	Nkombo	698 (89.5)		638 (81.8)		419 (53.7)		27 (3.5)	
Consistency of stool			0.05		0.02		0.62		0.82
	Formed	49 (89.1)		46 (83.6)		29 (52.7)		0	
	Soft	3817 (77.6)		3274 (66.6)		2457 (50.0)		93 (1.9)	
	Loose	6 (100.0)		6 (100.0)		2 (33.3)		0	
	Watery	13 (76.5)		12 (70.6)		7 (41.2)		0	
Stunting status (HAZ)			<0.001		<0.001		<0.001		0.04
	Non stunted	2569 (75.6)		2203 (64.8)		1638 (48.2)		54 (1.6)	
	Stunted	1316 (82.3)		1135 (71.0)		857 (53.6)		39 (2.4)	
Wasting status (BAZ)			0.002		<0.001		0.13		0.87
	Not wasted	3672 (78.2)		3175 (67.6)		2357 (50.2)		87 (1.9)	
	wasted	213 (70.5)		163 (54.0)		138 (45.7)		6 (1.9)	

**Table 3:** Negative binomial regression model for factors associated with eggs count/gram.

Variables		Hookworms			*Ascaris lumbricoides*			*Trichirus trichiura*		
		β (S.E)	95% CI	*p*	β (S.E)	95% CI	*p*	β (S.E)	95% CI	*p*
Sex	Male	1			1			1		
	Female	1.14 (0.24)	0.75–1.73	0.51	0.92 (0.03)	0.85–1.00	0.06	0.94 (0.03)	0.88–1.00	0.08
Age	5–9 years	1			1			1		
	10–15 years	1.45 (0.37)	0.87–2.4	0.15	1.06 (0.04)	0.97–1.16	0.15	1.13 (0.04)	1.05–1.21	0.001
District	Rubavu	1			1			1		
	Rutsiro	0.44 (0.22)	0.16–1.18	0.1	0.54 (0.03)	0.48–0.61	<0.001	0.09 (0.006)	0.07–0.10	<0.001
	Nyamasheke	2.00 (0.56)	1.15–3.48	0.01	0.28 (0.018)	0.25–0.32	<0.001	0.23 (0.115)	0.21–0.25	<0.001
	Rusizi	2.10 (0.57)	1.23–3.58	0.01	0.67 (0.033)	0.61–0.74	<0.001	0.63 (0.025)	0.59–0.68	<0.001
Stunting	Not stunted	1			1			1		
	Stunted	1.30 (0.29)	0.84–2.01	0.23	1.17 (0.05)	1.08–1.28	<0.001	1.10 (0.041)	1.02–1.19	0.006
Wasting	Not wasted	1			1			1		
	Wasted	1.10 (0.46)	0.47–2.53	0.82	1.02 (0.09)	0.86–1.21	0.79	0.91 (0.071)	0.78–1.06	0.26

S.E = standard error, CI = confidence interval.

**Table 4:** Univariate and multivariate logistic regression analysis of the factors associated with any STH infections.

Variables		Univariate Analysis			Multivariate Analysis		
		cOR	95% CI	*p*-Value	aOR	95% CI	*p*-Value
Sex	Female	1		<0.001	1		0.012
	Male	1.27	1.11–1.46		1.21	1.04–1.40	
Age categories	5–9 years	1		0.53			
	10–15 years	1.04	0.91–1.21				
District	Rutsiro	1			1		
	Nyamasheke	1.28	1.07–1.53	0.008	1.23	1.02–1.48	0.029
	Rusizi	6.62	5.31–8.25	<0.001	5.94	4.75–7.44	<0.001
	Rubavu	9.54	7.69–11.84	<0.001	9.5	7.64–11.81	<0.001
School	Sure	1					
	Rambo	11.55	8.30–16.07	<0.001			
	Rubona	8.53	6.37–11.42	<0.001			
	Rusororo	1.03	0.79–1.36	0.81			
	Buhokoro	1.37	1.06–1.77	0.02			
	Mukoma	1.23	0.94–1.60	0.13			
	Bugumira	5.9	4.24–8.21	<0.001			
	Nkombo	7.37	5.46–9.96	<0.001			
Stunting	Non stunted	1			1		0.001
	Stunted	1.5	1.30–1.75	<0.001	1.34	1.13–1.58	
Wasted	Non wasted	1			1		1
	Wasted	0.67	0.52 -0.86	0.002	1	0.76–1.32	
Schistosomiasis	No	1	1.48–3.14	<0.001	1		0.001
	Yes	2.16			1.95	1.31–2.90	

Due to the co-linearity of the school and district, the variable school was not included in the adjusted model. cOR = crude odds ratio, aOR = adjusted odds ratio, CI = confidence interval.

**Table 5:** Univariate and multivariate logistic regression analysis of the factors associated with soil-transmitted helminths species.

Variables		Hookworms				*Ascaris lumbricoides*				*Trichirus trichiura*			
		Univariate Analysis		Multivariate Analysis		Univariate Analysis		Multivariate Analysis		Univariate Analysis		Multivariate Analysis	
		OR (95% CI)	*p*	OR (95% CI)	*p*	OR (95% CI)	*p*	OR (95% CI)	*p*	OR (95% CI)	*p*	OR (95% CI)	*p*
Sex	Female	1				1		1		1		1	
	Male	0.93 (0.62–1.41)	0.74			1.15 (1.04–1.30)	0.009	1.13 (1.00–1.27)	0.05	1.17 (1.04–1.31)	0.011	1.12 (0.97–1.29)	0.11
Age	5–9 years	1		1		1		1		1			
	10–15 years	1.63 (0.99–2.68)	0.06	1.46 (0.88–2.43)	0.14	1.03 (0.91–1.16)	0.66			1.1 (0.96–1.24)	0.18		
District	Rutsiro	1		1		1		1		1		1	
	Rubavu	2.17 (0.82–5.72)	0.12	2.22 (0.84–5.86)	0.11	2.18 (1.84–2.57)	<0.001	2.19 (1.85–2.59)	<0.001	24.65 (19.90–30.54)	<0.001	24.76 (19.96–30.72)	<0.001
	Nyamasheke	4.47 (1.72–11.63)	0.002	4.50 (1.73–11.70)	0.002	0.50 (0.42–0.61)	<0.001	0.50 (0.42–0.61)	<0.001	3.06 (2.51–3.74)	<0.001	3.08 (2.52–3.76)	<0.001
	Rusizi	5.07 (1.99–12.96)	0.001	4.72 (1.84–12.13)	0.001	1.42 (1.19–1.69)	<0.001	1.35 (1.13–1.62)	0.001	14.20 (11.48–17.57)	<0.001	13.57 (10.94–16.83)	<0.001
School	Sure	1				1				1			
	Rambo	2.95 (0.66–13.25)	0.16			2.84 (2.22–3.62)	<0.001			25.08 (18.31–34.36)	<0.001		
	Rubona	2.30 (0.51–10.41)	0.28			1.93 (1.52–2.44)	<0.001			20.22 (15.09–27.10)	<0.001		
	Rusororo	1.38 (0.23–8.30)	0.73			1.10 (0.84–1.45)	0.49			0.82 (0.59–1.12)	0.211		
	Buhokoro	6.36 (1.47–27.58)	0.01			0.54 (0.41–0.70)	<0.001			2.87 (2.18–3.79)	<0.001		
	Mukoma	4.17 (0.91–19.15)	0.07			0.52 (0.39–0.69)	<0.001			2.64 (1.98–3.51)	<0.001		
	Bugumira	4.25 (0.93–19.51)	0.06			1.42 (1.09–1.85)	0.01			12.01 (8.75–16.48)	<0.001		
	Nkombo	7.23 (1.71–30.54)	0.01			1.54 (1.21–1.96)	<0.001			13.35 (10.00–17.82)	<0.001		
Stunting	Non stunted	1		1		1		1		1		1	
	Stunted	1.55 (1.02–2.35)	0.04	1.28 (0.83–1.99)	0.26	1.24 (1.10–1.40)	<0.001	1.22 (1.07–1.38)	0.002	1.33 (1.17–1.51)	<0.001	1.22 (1.04–1.42)	0.01
Wasted	Non wasted	1				1				1		1	
	wasted	1.07 (0.47–2.48)	0.87			0.84 (0.66–1.05)	0.13			0.56 (0.44–0.71)	<0.001	0.91 (0.69–1.20)	0.49
Schistosomiasis	No	1		1		1		1		1		1	
	Yes	3.00 (1.65–5.47)	<0.001	2.28 (1.24–4.19)	0.008	0.84 (0.66–1.05)	0.13	1.01 (0.79–1.13)	0.93	2.40 (1.74–3.31)	<0.001	2.40 (1.74–3.31)	<0.001

**Table 6:** Sociodemographic and baseline characteristics of school children (*n* = 4998).

Characteristics		*n*	%
Sex	Male	2344	46.9
	Female	2654	53.1
Age (Years)	5–9 years	1533	30.7
	10–15 years	3465	69.3
Stunting (HAZ) ^a^	Non stunted	3399	68.0
	Stunted	1599	32.0
Wasting (BAZ) ^b^	Not wasted	4696	94.0
	wasted	302	6.0
District	Rubavu	1807	36.2
	Rutsiro	846	16.9
	Nyamasheke	1081	21.6
	Rusizi	1264	25.3
**District**	**Schools**		
Rubavu	Rambo	831	16.6
	Rubona	976	19.5
Rutsiro	Rusororo	441	8.8
	Sure	405	8.1
Nyamasheke	Buhokoro	588	11.8
	Mukoma	493	9.9
Rusizi	Bugumira	484	9.7
	Nkombo	780	15.6

^a^ HAZ = height for age z-scores, ^b^ BAZ = BMI for age z-scores.
